# Evaluation of Availability, Prices, and Affordability of Selected Essential Medicines in Balochistan, Pakistan

**DOI:** 10.3389/ijph.2022.1604375

**Published:** 2022-07-06

**Authors:** Murad Bibi, Noman Ul Haq, Abdul Kareem, Habib Ullah, Nizam Baloch, Gulalai Rehman, Aqeel Nasim

**Affiliations:** ^1^ Department of Pharmacy Practice, University of Balochistan, Quetta, Pakistan; ^2^ Balochistan Institute of Nephrology Urology Quetta BINUQ, Quetta, Pakistan

**Keywords:** affordability, WHO, price, availability, essential medicine, Pakistan

## Abstract

**Objectives:** The study aimed to evaluate the availability, prices, and affordability of selected essential medicines in Balochistan, Pakistan.

**Methods:** Cross-sectional research was conducted in several cities of Balochistan, Pakistan, using the World Health Organization/Health Action International methodology to assess the availability and cost of 50 originator brand (OB) and lowest priced generic (LPG) drugs. The medicine costs were compared to international reference prices (IRPs) to calculate the median price ratio. The daily wage of the lowest paid unskilled government employee was used to determine affordability.

**Results:** The mean availability was low for OBs (9.8%) and fairly high (49.4%) for LPGs. The OBs and LPGs’ mean availability in the private sector were fairly high, 51.8% and 42.6%, respectively. It was surprising to see that Balochistan’s public sector has only 24.3% of the National Essential Medicine List when the medicines on this list are supposed to be adequately available.

**Conclusion:** The standard treatment cost with OBs is steep, exceeding the minimum daily wage. Treatment with LPG medications seems affordable. Furthermore, essential LPG medicines are economical when used solely for medication therapy.

## Introduction

Access to medicines has helped enhance patient health outcomes and lower mortality rates [1]. Non-availability and excessive medicine pricing are two significant barriers to accessing medicines. In the past decades, little progress has been made to improve the availability essential medicines that remain unaffordable and unavailable to many poor patients, who cannot even afford their daily needs. Essential medicines satisfy the priority healthcare needs of the population. Their policies are crucial to promoting health and achieving sustainable development. The Sustainable Development Goal 3.8 mentions the importance of “access to safe, effective, quality, and affordable essential medicines and vaccines for all” [2]. To ensure access to medical treatment and achievement of their ultimate objective of improving their quality of life, public and private-sector patients must have sufficient access to medications [3].

The World Health Organization (WHO) estimated that approximately 38% of generic drugs were available in the private sector within low- and middle-Income countries, including Pakistan, and were estimated to cost almost three times more than the worldwide benchmark pricing. However, private institutions had approximately 60% of generic medicines that were available and better, but these were six times more expensive than the international prices [4]. The total cost of healthcare depends substantially on drug prices, predicted as 11%–16% in developed nations, including Pakistan, and up to 32%–42% cost of healthcare in nearly all emerging republics [5].

Within this context, medical stores and community pharmacies have recently become the primary source for purchasing medicines in developing countries for most of the population [6]. Regardless of the regulatory authorities, the market for medicine supply is growing rapidly in developing countries. This reduces the likelihood of most people having restricted access to medication [7]. However, a positive approach was that most medicines were available to the public in these privately owned medical stores or pharmacies that fill the supply gap [8].

In collaboration with Health Action International (HAI), the WHO produced a standard methodology relating to access to essential medicines, which have been extensively used in different studies. Many developing countries have developed essential medicine lists, but it is not enough to impact the availability of these medications to the population. It was estimated that almost half of the world’s population still lacks access to life-saving medicines [9]. Many acute and chronic diseases are still untreatable, and patients cannot afford to treat such illnesses. This ultimately enhances the overall burden of diseases and healthcare costs, and the prime reason for that is the low availability of medicines in these countries. Although the prices of essential generic medicines are lower in some countries, they are not affordable to the majority of the population [2].

According to the Alma Ata Declaration concerning access to medicine, there is a reasonable gap in Pakistan for the availability of essential medicine. In Pakistan, the government is responsible for providing free medication to government hospitals where a patient in need can come for treatment. However, the unavailability of such medicines in government primary, secondary, and tertiary care hospitals might induce patients to buy essential medicines from private medical stores/pharmacies where they have to pay the cost with their own money [10].

After extensive literature research, it was found that only a few studies have been reported regarding availability, affordability, and essential medicines in Pakistan and no study was done in Balochistan. Therefore, this study was designed to assess the availability, affordability, and essential medicines in Balochistan.

## Methods

The WHO/HAI methodology was used to undertake cross-sectional descriptive research in many cities across Balochistan, Pakistan and the data gathered were from both government and private healthcare facilities. The study was conducted from December 2019 to February 2020.

### Medicine Selection

As per the WHO/HAI guidelines, each country must select a list of main and complementary medicines based on local needs and the prevalence of diseases. The core list is of the most efficacious, safe, and cost-effective medications for priority illnesses that should be provided in a basic healthcare system. The study included 50 drugs that met most of the population’s healthcare demands.

### Selection of Medicine Outlets

Data were obtained from nine districts of Balochistan province of Pakistan, which were Quetta, Pishin, Mastung, Kalat, Khuzdar, Turbat, Sibi, Dera Murad Jamali, and Zhob. The main public hospital was chosen in each district. Subsequently, within 10 km of each public medicine institute, a licensed pharmacy/medicine store was also visited to check the availability of the medicines mentioned above.

### Data Collection

Health facilities/institutions were approached to gather drug pricing and availability data. Data were acquired with the same dosing regimen in all facilities. Each drug’s strength and pack size was recorded for local and worldwide comparisons. For each medicine, data were collected for the originator brand (OB) (international brand product) and the lowest priced generic (LPG) equivalent, that is, any product other than the originator brand that includes the same active ingredient. Price data were gathered from government and private sector patient expenses and public procurement prices.

### Data Analysis

Medicine Prices: Individual medication prices were stated in two ways

(1) The medicine’s median unit costs, and
(2) The ratio to the international reference price (IRP) is represented as the median price ratio (MPR) to allow worldwide comparison.

The MPR was derived by multiplying the IRP by the drug’s median local price. Compared to the IRP, the MPR was assessed regarding how much greater or lesser it was. With an MPR of 2, the local medicinal price is twice that of the IRP.

The MPR can be calculated using the following formula.

Median price ratio (MPR) = median local unit price/international reference unit price.

The Management Sciences for Health handbook can assess IRP (MSH 2014). The procurement and patient price data were displayed using the median of MPRs, the 25th and 75th percentile MPRs, and the minimum and maximum MPRs.

The ideal value for MPR was used to represent acceptable local price ratios:

• Public-sector procurement prices MPR 1
• Public-sector patient pricing MPR 1.5
• Patient prices in private pharmacies MPR 2

If the MPR for patient pricing in public and private sectors is twice or more than the IRP, the prices will become unaffordable. The purchase price is used to determine the efficiency of the purchasing method. The purchasing mechanism is particularly efficient and effective if the median is less than 1.00 MPR. In contrast, MPRs greater than one can raise questions regarding purchasing efficiency [11].

### Medicine Availability

Medicine availability was documented as follows:

• Absent: 0% of medicines;
• Low: < 50% of medicines;
• Fairly high: 50%–80% of medicines;
• High: > 80% of medicines.

### Medicine Affordability

The number of working days (daily salary) of an inexperienced public servant with the lowest wages, permitting them to acquire a standard treatment plan for common illnesses with selected drugs, was utilized to evaluate the affordability of medications. Patients’ affordability of conventional treatments for various ailments was determined using the pay of the lowest paid government employee of 17000 Pakistani Rupees per month for 2019–20.

The expense of a complete course of treatment for acute diseases and the expense of a 1-month chronic disease treatment course were considered. In the public and private sectors, 7 days of medical provision for an acute illness or 1 month for chronic illness would be considered affordable. Standard treatment costs 1.5 days’ pay (median) for OBs and 0.8 days’ wages (median) for LPG medications.

## Results

### Originator Brands and Lowest Priced Generic Medicine Availability in Public and Private Sector

Table 1 shows the availability of OB and LPG medicine in the public and private sectors.

**TABLE 1:** Availability of originator brand and lowest priced generic medicines in the public and private sector.

Medicine name	OB		LPG	
	Public (%)	Private (%)	Public (%)	Private (%)
Acyclovir	40	80	20	20
Albendazole	10	70	70	60
Allopurinol	20	80	10	10
Amiloride	0	10	20	0
Amoxicillin	10	100	80	60
Atenolol	20	70	70	40
Atorvastatin	0	20	80	80
Atracurium	0	20	40	70
Azathioprine	20	70	40	60
Captopril	0	10	40	50
Carbamazepine	20	90	20	10
Ceftriaxone	0	80	80	90
Ciprofloxacin	0	80	100	60
Clarithromycin	0	60	80	80
Clopidogrel	20	90	60	60
Dexamethasone	30	90	70	40
Diazepam	30	90	30	10
Diclofenac Sodium	0	60	40	20
Enalapril	0	30	40	60
Fluoxetine	0	0	30	40
Folic Acid	20	100	70	0
Furosemide	20	100	60	0
Glibenclamide	0	30	30	80
Haloperidol	0	10	50	40
Hydralazine	20	80	20	0
Ibuprofen	20	100	80	90
Isoniazid	0	10	20	0
Labetalol	20	80	10	0
Loratadine	30	80	50	30
Losartan Potassium	0	30	90	80
Metformin	20	100	70	70
Methylprednisolone	0	30	10	10
Metoclopramide	0	30	50	70
Metronidazole	0	40	90	60
Miconazole	0	10	40	80
Nalbuphine	20	80	30	10
Nystatin	0	40	90	70
Omeprazole	20	100	90	90
Ondansetron	20	80	50	0
Paracetamol Susp	20	100	100	90
Prednisolone	0	10	40	50
Primaquine	0	10	10	10
Propranolol	20	90	40	20
Sodium Stibogluconate	0	0	20	10
Spironolactone	0	20	60	70
Streptokinase	0	0	30	0
Sulfasalazine	0	10	40	70
Tetracycline	0	30	10	60
Tranexamic Acid	0	40	90	60
Verapamil	0	10	30	10

OBs, Originator brands.

LPGs, Lowest priced generics.

#### Public Sector

OBs were available in the public sector (44.0%) and availability of LPGs was higher as compared to OBs.

#### Private Sector

OBs were available in the private sector (96.0%). Moreover, 9/50 LPGs were not available in the private sector.

### Availability of Medicine in Public and Private Sectors

#### Public Sector

No medicine was identified in most public sector facilities (high availability >80%). Most of the drugs surveyed in public sector facilities were unavailable (0% availability) (Table 2).

**TABLE 2:** Availability of medicines in public and private sectors.

Availability	Range	Public sector		Private sector	
		OB	LPG	OB	LPG
Absent	0%	Ceftriaxone, Ciprofloxacin, Clarithromycin, Isoniazid, Nystatin Metronidazole, Sodium Stibogluconate, Primaquine, Methyl prednisolone, Transmit Acid, Deferoxamine, Losartan Potassium, Verapamil, Enalapril, Streptokinase, Atorvastatin, Miconazole, Amiloride, Spironolactone, Metoclopramide, Sulfasalazine, Glibenclamide, Atracurium, Tetracycline, Prednisolone, Haloperidol, Fluoxetine, Timolol	—	Sodium Stibogluconate, Deferoxamine, Streptokinase, Fluoxetine	Ondansetron, Isoniazid, Folic Acid, Deferoxamine, Labetalol, Hydralazine, Streptokinase, Amiloride
Low	<50%	Nalbuphine, Dexamethasone, Diazepam, Ondansetron, Loratadine, Carbamazepine, Albendazole, Amoxicillin, Acyclovir, Paracetamol, Ibuprofen, Propranolol, Azathioprine, Allopurinol, Folic Acid, Clopidogrel, Labetalol, Hydralazine, Furosemide, Omeprazole, Metfornin, Atenolol,	Primaquine, Allopurinol, Methylprednisolone, Labetalol, Tetracycline, Carbamazepine, Isoniazid, Acyclovir, Sodium Stibogluconate, Deferoxamine, Hydralazine, Amiloride, Nalbuphine, Diazepam, Verapamil, Streptokinase, Glibenclamide, Fluoxetine, Propranolol, Azathioprine, Enalapril, Miconazole, Sulfasalazine, Atracurium, Prednisolone, Timolol	Isoniazid, Primaquine, Verapamil, Miconazole, Amiloride, Sulfasalazine, Prednisolone, Haloperidol, Timolol, Atorvastatin, Spironolactone, Atracurium, Methylprednisolone, Losartan Potassium, Enalapril, Metoclopramide, Glibenclamide, Tetracycline, Nystatin, Metronidazole, Transmic Acid,	Nalbuphine, Diazepam, Carbamazepine, Sodium Stibogluconate, Primaquine, Allopurinol, Methyl prednisolone, Verapamil, Acyclovir, Propranolol, Loratadine, Dexamethasone, Haloperidol, Fluoxetine, Atenolol,
Fairly High	50%–80%	—	Ondansetron, Loratadine, Metoclopramide, Haloperidol, Clopidogrel, Furosemide, Spironolactone, Dexamethasone, Albendazole, Folic Acid, Metformin, Atenolol, Amoxicillin, Ceftriaxone, Clarithromycin, Ibuprofen, Atorvastatin,	Clarithromycin, Albendazole, Azathioprine, Atenolol, Nalbuphine, Ondansetron, Loratadine, Ceftriaxone, Ciprofloxacin, Acyclovir, Allopurinol, Labetalol, Hydralazine,	Prednisolone, Timolol, Albendazole, Amoxicillin, Ciprofloxacin, Metronidazole, Azathioprine, Clopidogrel, Tranexamic Acid, Enalapril, Tetracycline, Nystatin, Spironolactone, Metoclopramide, Sulfasalazine, Metformin, Atracurium, Clarithromycin, Losartan Potassium, Atorvastatin, Miconazole, Glibenclamide,
High	>80%	—	Nystatin, Metronidazole, Tranexamic Acid, Losartan Potassium, Omeprazole, Ciprofloxacin, Paracetamol	Dexamethasone, Diazepam, Carbamazepine, Propranolol, Clopidogrel, Amoxicillin, Paracetamol, Ibuprofen, Folic Acid, Furosemide, Omeprazole, Metformin	Ceftriaxone, Paracetamol Ibuprofen, Omeprazole

#### Private Sector

Only 4 medicines, sodium stibogluconate, deferoxamine, streptokinase, and fluoxetine, were unavailable in the private sector (0% availability), whereas the remaining 46 medicines were available (Table 2).

### National Essential Medicine List at Various Healthcare Levels

The availability of medicines in the government sector was assessed. As per Pakistan’s National Essential Medicine List (NEML), only medicines intended to be provided at every level of care were included. However, it was surprising to see that Balochistan’s public sector has only 24.3% of the NEML when the medicines on this list are supposed to be adequately available. Conversely, the private sector was much better on account of NEML availability, as shown in Table 3.

**TABLE 3:** National essential medicine at various healthcare levels.

	Public sector (%)	Private sector (%)
National essential medicine	24.3	73.6

### Prices of Medicines in Public and Private Sectors

#### Procurement Prices in the Public Sector

Public procurement costs were known for 22 of the 50 drugs purchased as OBs and 46 as LPGs. LPGs were bought for 2.85 times the optimal value of MPRs, whereas OBs were bought for 3.96 times the ideal value of MPRs. The MPRs of LPGs (0.32–7.86) and OBs (0.55–6.87) varied widely among all of the drugs studied (Table 4).

**TABLE 4:** Procurement prices in the public sector.

Total medicines (*n* = 50)	MPR (median)	25% MPR	75% MPR	Least MPR	Extreme MPR
OB	3.96	1.98	5.94	0.55	6.87
LPG	2.85	1.425	4.275	0.32	7.86

#### Patient Prices in Public and Private Sectors

The analysis includes medicines from the public and private institutes to evaluate price. Patient spending for LPGs in the private market was 5.5 times higher than in the public sector. OBs were also 1.4 times more expensive in the private sector (Table 5).

**TABLE 5:** Patient prices in public and private sectors.

Type	Government sector patient prices (median MPR)	Private sector patient prices (median MPR)	% Difference (%)
OB	4.8	8.7	66
LPG	1.15	7.3	524

### Affordability of Standard Treatment Regimens

Ciprofloxacin (3.5), clarithromycin (2.6), carbamazepine (1.4), and ceftriaxone (1.9) were all regarded as unaffordable medications that exceeded the minimum daily wage. Nonetheless, treatment with LPG medications seemed affordable, costing the same as or less than a day’s work (Supplementary File S1).

### International Reference Price and Median Price Ratios

#### Patient Prices in the Private Sector

Patient pricing MPRs in the private institute for eight medications in Pakistan (Balochistan Province), (Lahore City), and three other countries. (India, China, and Afghanistan) were utilized. Patient prices for amoxicillin, ciprofloxacin, captopril, diclofenac sodium, diazepam, and omeprazole were the highest in Pakistan, according to the findings. Pakistan had lower patient pricing for ciprofloxacin OBs than Afghanistan (Supplementary File S2).

## Discussion

Only one survey has been conducted in Pakistan using the WHO/HAI methodology [12]. However, this standardized methodology has not been used in Balochistan, making this study novel in its scope when applied to the largest province of Pakistan. This study examined the costs of medicines, their availability, and affordability to the Pakistani people, and the prices were compared to those worldwide using the WHO/HAI handbook [13].

### Availability of Surveyed Medicines

According to our study, Pakistan’s private sector has better access to critical medications than the public sector. This is consistent with research performed in other countries with the most private-sector availability [14, 15]. The absence of available medications in the public sector is due to insufficient government financing for the healthcare sector. Furthermore, research in six low- and middle-income countries revealed that overall medicine provision in government departments was considerably lower when compared to private sectors [16].

This study demonstrated that OB and LPG essential drugs were overpriced at private sector pharmacies in Quetta. In addition, current research has been conducted for a long time (5–6 months) in different medical facilities and has, therefore, provided a realistic assessment of the general situation patients face daily.

Compared to private health facilities, the overall availability of OB medications was meager in public health facilities. In contrast, LPG was more available than OB but remained very low in both sectors. Also, for the treatment of common diseases, LPGs were available, but OBs were not. The survey showed that patients pay significantly more for the purchase of OB in all industries than cheaper generic drugs. This practice was also seen in a study conducted in Sudan, where the author has stated the same results that the customers or patients have to pay more for an OB than an LPG medication [17]. Another study supported this argument that prices for generic drugs in public and private sectors were significantly higher than expected if the purchase and distribution were effective and doctor appointments were reasonably priced [18].

The findings show that, on average, the government procurement agency purchases the cheapest generic drugs. Compared to OB purchases, the government procurement agency purchases LPGs regardless of price. These findings agree with earlier research findings, which suggest that the government procurement agency purchases OBs efficiently, albeit at exorbitant prices. As a result of add-on charges in the public sector distribution chain, these drugs are then sold on to patients at a price almost 19.6% higher than the purchase price [19].

When we compared our findings to those of other middle-income nations (Egypt, India, Lebanon, and China), Pakistan came in third, trailing behind only India and Lebanon regarding medicine availability, despite the small sample size making these differences seem insignificant. In contrast, a study in China’s rural areas revealed that LPG is scarce in the public and private sectors (39% and 44%, respectively) [20].

Low- and middle-income nations account for one-third of the global population, but they are among the most underserved in inexpensive vital medicines. Patients are frequently forced to purchase medications from private facilities, which charge exorbitant fees.

### Prices of Surveyed Medicines

The analysis found that the perceived sector’s LPG procurement prices were higher than the IRPs. LPGs were purchased for 2.85 times the ideal MPR value, whereas OBs were purchased for 3.96 times the appropriate MPR value. This finding was supported by the fact that LPG procurement prices were 13% higher than IRPs. Conversely, OB procurement prices were 5.5 times higher than IRPs. This implies that the public sector’s LPG and OB procurement prices are greater than IRPs [11]. Likewise, a Chinese investigation discovered that the cost of buying OB was more than that of buying LPG [20]. Another Sri Lankan study discovered that the public sector’s LPG purchase price was somewhat lower than that of IRPs (MPR 0.82) [21].

Patient prices for OB and LPG in the private sector were roughly three to four times higher than those in IRPs. As a result, private pharmacies’ OB and LPG prices are regarded as excessive. Additionally, patients in the private sector pay more for OB than LPG. This finding is comparable to a study conducted in Swaziland, which found that patient prices for OBs in the private sector were 4.7 times higher than LPG prices [21]. Another study examined patient prices in the public and private sectors, finding that patient prices for LPGs and OBs in the private sector were 554% and 68% higher than those in the public sector [22]. Similar findings revealed that public procurement prices were quite competitive, with medians well below the IRPs [23].

### Affordability of Surveyed Medicines

The daily income of the lowest paid unskilled government worker is used to compute the cost of a single course of therapy for specific diseases [24]. The number of days the lowest paid unskilled government employee would have to work to pay for one treatment course for acute disease, or 1 month’s supply of medicines for a chronic condition, was used to evaluate affordability. This study’s findings revealed that some diseases, such as epilepsy treated with carbamazepine, and adult respiratory infection treated with ciprofloxacin or ceftriaxone injection in the private sector, require more than a day’s income (1.1–3.5 days) to afford the required medicine. Another research reveals that various disorders, including high blood pressure managed with bisoprolol or captopril, hypercholesterolemia, arthritis, ulcer managed with omeprazole or ranitidine, and adult respiratory infection treated with LPGs or OBs, necessitate more than 1 day of income (1.1–4.4 days) [11].

Patients could not afford antiepileptic drugs, according to this study. Additionally, other antiepileptic medications were unaffordable, requiring 3–12 days of employment. Considering that epilepsy is still a stigmatized condition associated with poverty when sufferers are unable to work consistently [25].

Injection therapy with ceftriaxone produced similar results. Ceftriaxone has the lowest affordability across all industries, according to the author. This could be due to the need for administrative costs and other expenses (such as syringes and water for injection.) Even though they do not administer the medicine directly to the patient, the cost is high in retail pharmacies [15].

In the private sector, conventional treatment for anxiety, allergy, pain, and protozoal infections, administered by LPGs or OBs, is affordable, costing only 1 day’s wages or less for the lowest paid unskilled government employee. This was similar to a study in which the author demonstrated that standard treatment of conditions such as asthma, diabetes, depression, adult respiratory infection treated with amoxicillin, pediatric respiratory infection, anxiety, pain/inflammation in children, and epilepsy treated by LPGs or by OBs are affordable and cost only a day’s income (or less) of the lowest paid unskilled government worker [11]. According to a recent study in Tanzania, therapy cost is the most critical factor in determining whether patients receive suitable or inappropriate care. An increase in the affordability of pharmaceuticals could help enhance treatment [26].

In general, retailing adds to the expense of doing business. Higher costs at various levels of the supply chain might increase costs and jeopardize patients’ capacity to afford medications. Prices can be reduced due to this if the supply chain is well organized.

### Conclusion

The mean availability of OBs was low (9.8%) and fairly high (49.4%) for LPGs. The OBs and LPGs’ mean availability in the private sector were fairly high, 51.8% and 42.6%, respectively. It was surprising to see that Balochistan’s public sector has only 24.3% of the NEML when the medicines on this list are supposed to be adequately available. Conversely, the private sector was much better on account of NEML availability. The cost of standard treatment with OBs was unaffordable. Antibiotics (ciprofloxacin, clarithromycin, and ceftriaxone) and treatment courses for seizures (carbamazepine) were unaffordable medications that exceeded the minimum daily wage. Nonetheless, treatment with LPG medications seemed affordable, costing the same as or less than a day’s work. Furthermore, essential LPG medicines were found economical when used solely as medication therapy. The study emphasizes existing concerns in Balochistan regarding the pricing, availability, and affordability of essential medicines for the treatment of chronic medical conditions. It has backed the necessity for policy development regarding pricing regulations and mark-up control, in order to increase availability, lower prices, and enhance affordability.

### Recommendations

This study’s findings indicate that pricing policies can be implemented to make drugs more accessible and available, as well as stimulate generic prescription, distribution, and substitution. The generic policy in acquiring drugs in the public sector must be maintained.

### Strengths and Limitations

#### Strengths

This study’s advantage is its use of the WHO guidelines, which is the operational package for measuring, monitoring, and evaluating countries’ pharmaceutical situations. Furthermore, the study has an adequate sample size. Consequently, this study’s results can be applied to various populations globally.

#### Limitations

This study’s limitation is that the minimum wage was utilized to evaluate whether conventional therapy or particular medicines for a given disease were affordable. However, many patients in the research region may earn less, and many dependents may rely on one person’s income.

## Data Availability

The data used to support the findings of this study are included in the article.

## Abbreviations

HAI, Health Action International; IRPs, international reference prices; LPG, lowest priced generic; MDGs, millennium development goals; MPR, median price ratio; NEM, national essential medicine list; OB, originator brand; WHO, World Health Organization.
