# Supplementary material for: Evaluation of Availability, Prices, and Affordability of Selected Essential Medicines in Balochistan, Pakistan
Source: Int J Public Health. 2022 Jul 6;67:1604375. doi: 10.3389/ijph.2022.1604375 (PMC9296778; doi:10.3389/ijph.2022.1604375)
Supplement: Supplementary file 1 [file Table1.docx]

**Supplementary File. 01**

**Affordability of standard treatments in private retail pharmacies by lowest paid unskilled government worker**

| **S#** | **Medicine name and Strength** | **Dosage Form** | **Disease Condition** | **Total Duration**  **(Days)** | **Units required for treatment** | **Median Treatment Price (PKR)** | **Day’s wage for treatment** |
| --- | --- | --- | --- | --- | --- | --- | --- |
|  | Diazepam 5mg | Tab | Anxiety | 07 | 07 | 19.2 | 0.1 |
|  | Loratadine 10mg | Tab | Allergy | 30 | 15 | 85.2 | 0.4 |
|  | Amoxicillin 500mg | Cap | Infection | 7 | 14 | 91.0 | 0.5 |
|  | Ceftriaxone 1gm | Inj | Infection | 1 | 14 | 450.0 | 1.9 |
|  | Ciprofloxacin 500mg | Tab | UTIs | 14 | 28 | 1263.64 | 3.5 |
|  | Clarithromycin 500mg | Tab | Bronchitis | 7 | 14 | 855.5 | 2.6 |
|  | Metronidazole 400mg | Tab | Protozoal Infection | 7 | 21 | 45.57 | 0.2 |
|  | Primaquine 15mg | Tab | Malaria | 14 | 15 | 30.3 | 0.25 |
|  | Paracetamol 120mg/5ml | Susp | Pain | As needed | 4 | 13.5 | 0.1 |
|  | Ibuprofen | Syp | Pain | As Needed | 3 | 45.0 | 0.3 |
|  | Carbamazepine 200mg | Tab | Epilepsy | 30 | 60 | 390.0 | 1.4 |
|  | Atenolol 50mg | Tab | Cardiovascular Diseases | 30 | 60 | 129.75 | 0.8 |

Affordability of standard treatments in private retail pharmacies by lowest paid unskilled government worker (Evaluation of Availability, Prices and Affordability of Selected Essential Medicines in Balochistan, Pakistan Balochistan, Pakistan, 2019-20)
