# Supplementary material for: Evaluation of Availability, Prices, and Affordability of Selected Essential Medicines in Balochistan, Pakistan
Source: Int J Public Health. 2022 Jul 6;67:1604375. doi: 10.3389/ijph.2022.1604375 (PMC9296778; doi:10.3389/ijph.2022.1604375)
Supplement: Supplementary file 2 [file Table2.docx]

**Supplementary File. 02**

**Patient prices in private sector**

|  | **Affordability (According to wage required per day)** | | | | | | | | | |
| --- | --- | --- | --- | --- | --- | --- | --- | --- | --- | --- |
| **Medicine Name** | **Pakistan**  **(Balochistan)**  **2019-20** | | **Pakistan**  **(Punjab)**  **2016-17** | | **India**  **(NCT Delhi)**  **2011** | | **China**  **(Shaanxi Province, September 2014** | | **Afghanistan**  **(2011)** | |
|  | **OB** | **LPG** | **OB** | **LPG** | **OB** | **LPG** | **OB** | **LPG** | **OB** | **LPG** |
| Amoxicillin 500mg | 2.5 | 0.5 | 0.4 | NA | NA | 0.8 | NA | 0.3 | 0.7 | 0.4 |
| Ciprofloxacin 500mg | 2.7 | 2.2 | 1.5 | NA | NA | 0.3 | NA | NA | 2.8 | 0.5 |
| Paracetamol  120 mg/5ml | 1.3 | 0.1 | 0.1 | NA | 0.1 | 0.1 | NA | NA | 0.2 | 0.1 |
| Captopril 25mg | 2.1 | 0.8 | 1.0 | NA | NA | 1.1 | NA | 0.0 | NA | 0.4 |
| Atenolol 50mg | 2.1 | 0.8 | 0.4 | NA | 0.4 | 0.4 | NA | NA | NA | 0.2 |
| Omeprazole 40mg | 3.5 | 1.1 | 3.2 | NA | NA | 0.5 | NA | NA | NA | 0.6 |
| Diclofenac 50mg | 1.1 | 0.7 | 0.7 | NA | 0.8 | 0.4 | NA | 0.9 | NA | 0.4 |
| Diazepam | 2.5 | 1.1 | NA | NA | 0.1 | 0.1 | NA | NA | NA | 0.0 |

Patient prices in private sector (Evaluation of Availability, Prices and Affordability of Selected Essential Medicines in Balochistan, Pakistan Balochistan, Pakistan, 2019-20)
